# A German Version of the Staff Attitude to Coercion Scale. Development and Empirical Validation

**DOI:** 10.3389/fpsyt.2020.573240

**Published:** 2021-01-18

**Authors:** Simone A. Efkemann, Matthé Scholten, Ronald Bottlender, Georg Juckel, Jakov Gather

**Affiliations:** ^1^Department of Psychiatry, Psychotherapy and Preventive Medicine, LWL University Hospital, Ruhr University Bochum, Bochum, Germany; ^2^Institute for Medical Ethics and History of Medicine, Ruhr University Bochum, Bochum, Germany; ^3^Klinik für Psychiatrie und Psychotherapie, Klinikum Lüdenscheid, Märkische Klinken, Lüdenscheid, Germany

**Keywords:** psychiatry, mental health care, coercive measures, attitudes research, test adaptation, compulsory treatment, involuntary admission

## Abstract

**Background:** Individual staff factors, such as personality traits and attitudes, are increasingly seen as an important factor in the reduction of coercion in mental health services. At the same time, only a few validated instruments exist to measure those factors and examine their influence on the use of coercion.

**Aim:** The present study aimed to develop and validate a German version of the Staff Attitude to Coercion Scale (SACS).

**Methods:** The original English version of the SACS published was translated into German. Subsequently, it was empirically validated on a sample of *N* = 209 mental health professionals by conducting an exploratory factor analysis.

**Results:** The three-factor structure in the original version of the SACS, consisting of critical, pragmatic and positive attitudes toward the use of coercion, could not be replicated. Instead, the German version revealed one factor ranging from rejecting to approving the use of coercion.

**Conclusion:** The SACS is one of the first instruments created to assess staff attitudes toward coercion in a validated way. The version of the instrument developed in this study allows for a validated assessment of those attitudes in German. Our results highlight the ethical importance of using validated measurements in studies on the role of staff factors in the reduction of coercion.

## Background

Strong efforts have been made in recent years to reduce the use of coercion in psychiatry (1, 2). These efforts have been driven by the firm ethical belief that coercive measures are *prima facie* morally problematic because they are associated with negative consequences for those affected (3). Against this background, the demand has been raised to reduce the use of coercion to an absolute minimum, and some even completely renunciate the use of all measures against a person's will, often with reference to the United Nations Convention on the Rights of Persons with Disabilities (4–11). Several guidelines (12, 13) and specific programs (14, 15) have been developed in the area of clinical psychiatry to reduce coercion.

It is essential to determine the underlying causes and justifications for the use of coercion in clinical situations to develop further innovative strategies to reduce coercive interventions in psychiatry. In this context, staff characteristics have recently been receiving more attention regarding their role in the clinical decision-making process and the use of coercion in mental health services (16, 17). Mental health professionals' attitudes have been examined in empirical studies, mostly in relation to their impact on the use of coercive interventions (18–22), such as mechanical restraint or seclusion (23–26). It is assumed that mental health professionals' cognitive and emotional attitudes toward coercion influence the way in which they decide and behave in certain situations and, consequently, influence the frequency and type of coercive interventions. Even though studies underline the relevance of the staff attitudes toward the use of coercion (18, 24), little is known about its precise impact on the actual use of coercive measures (27). Furthermore, only a few studies have addressed the question how staff attitudes evolve (28–30) and whether the latter can be modified (e.g., by means of training) (31–35).

The empirical investigation into the role of staff attitudes in the use of coercion is paramount from an ethical perspective because it yields important insights into the potential effectiveness of programs to reduce coercion. If it turns out that staff attitudes play a key role in the use of coercion, providing training for mental health professionals and achieving culture change might, for example, be more efficient in reducing coercion than the reform of mental health law and policy. From a theoretical point of view, the clarification of the aforementioned questions requires a clear and appropriate concept and operationalization of attitudes, which is applicable in the context of coercion in psychiatry. Attitudes toward coercion in studies on staff attitudes in psychiatry tend to be interpreted in a variety of terms, ranging from the appraisal of ethical or legal legitimacy, the degree of approval of coercive measures to self-reported preparedness to use coercion (19, 36, 37). Attitudes in psychology, are commonly divided into three components: A cognitive component, including thoughts and beliefs, an affective component, including feelings and emotions, and a behavioral component, including concrete actions (38). Attitudes can, thus, be understood as cognitive, emotional and behavioral dispositions that are, at least to some extent, under our voluntary control. It is assumed here that the cognitive and affective components have an impact on the behavioral component (39, 40). While the behavioral component is easily measurable, the measurement of the cognitive and affective proportions turns out to be challenging (41).

Furthermore, cognitive and emotional attitudes can be explicit or implicit (41). Measurements of implicit attitudes rely on the assumption that participants are often not consciously aware of certain beliefs or emotions or do not want to express them due to social desirability. Many tests for implicit attitudes use reaction time to respond to certain items as a variable to identify unconscious preferences or pre-judicial attitudes. The Implicit Association Test is a notable example. The validity of such tests has been questioned (42). Explicit measurements of the cognitive and affective components of attitudes are based mainly on self-reports in the form of agreements and disagreements with certain statements and, as such, address aspects of which people are consciously aware. An example of an instrument that measures explicit attitudes is the Attitudes to Containment Measures Questionnaire (43), which uses images to assess participants' approval of different kinds of coercive measures. Another questionnaire assesses nurses' attitudes toward and knowledge and practices of mechanical restraint (44). Various underlying concepts and definitions used in empirical studies and different scopes of coercion (i.e., specific coercive interventions or coercion in general) complicate the measurement of explicit attitudes and the comparison of research results. Consequently, many international studies have relied on either qualitative research designs or questionnaires that were developed or adapted for specific research questions and whose validity often remains unclear (23, 26, 29, 34, 45).

Regarding the Staff Attitude to Coercion Scale (SACS), Norwegian researchers developed and validated an instrument which facilitates a standardized and quantitative measurement of the cognitive component of mental health professionals' attitudes toward coercion in general (46, 47). Based on focus groups with mental health professionals, the researchers developed items for a questionnaire in the form of statements that represent certain beliefs about coercion. They created a self-report questionnaire with 15 items, which are assessed with a 5-point Likert scale ranging from total disagreement to total agreement. The principal component analysis seemed to reveal a three-factor structure. Based on this, the researchers proposed three independent types of attitudes toward coercion: A pragmatic attitude (coercion as care and security), a critical attitude (coercion as offending) and a positive attitude (coercion as treatment), with sufficient reliability for all three subscales (47). The subscales are scored as the sum of the corresponding items. After the development and validation of the SACS, the authors used the questionnaire to examine attitudes of mental health professionals and their influence on the use of coercive measures (48). They could show that there were differences in the attitudes between different staff members, but the actual use of coercive measures on different wards was not associated with staff attitudes on these wards. The original version was developed in Norwegian and used in the first studies published by Husum et al., but the items were translated from Norwegian to English through a validated process for the publication of the results from the validation study (Husum, personal communication, 2020). The SACS has been widely used in international studies, but it remains unclear whether the respective research teams used the published English translation of the original version or a (perhaps unpublished) validated further translation (31, 49–51). The SACS has also been used in studies from German-speaking countries (52–54), even though no validation of a German version has yet been published.

Against this background, the major aim of our study was, firstly, to develop and adapt the original version of the SACS into the German language and context. Secondly, an empirical validation of the instrument should examine its feasibility, reliability and validity. During the process, we reflected critically on conceptual and methodological aspects of the SACS and drew conclusions about the interpretation of results from studies relying on the SACS and future research on staff attitudes toward coercion.

## Methods

### Translation and Adaptation of the Instrument

The translation and adaptation of the SACS followed the guidelines of the International Test Commission (55) for the translation and adaptation of questionnaires. In a first step, the English items were translated by native German-speaking researchers. Mental health professionals were then asked for feedback on these items. Subsequently, all items were back translated by a bilingual researcher and an independent lay person raised bilingually in English and German. Regarding items with notable differences between the back translation and the original version, the German translation was further adapted with consultation of the bilingual researcher who was involved in the back translation. Afterwards, the final items were once again presented to different mental health professionals to receive feedback regarding linguistic and logical comprehensibility.

### Empirical Validation: Feasibility, Reliability, and Validity

After finishing the translation and adaptation of the original version of the SACS, the final German version was validated empirically with data assessed in three steps. The empirical validation was approved by the Research Ethics Committee of the Medical Faculty of the Ruhr University Bochum (Reg. No.: 17-6284). The validation was conducted as a developing process in which findings obtained at one stage determined the following steps at the next stage. Furthermore, important aspects (feasibility, reliability, and validity) were addressed at different stages of the validation. The options to assess validity and reliability were limited. The former were limited because no comparable measurements exist which could be used to assess criterion validity. The latter were limited because our data had to be collected anonymously, as a result of which we could not assess retest-reliability. Consequently, our examination of reliability and validity focused on internal consistency, face validity, and construct validity.

We performed a pretest before collecting the data to assess feasibility and face validity for the adapted version of the SACS. To this end, several professionals and researchers from various backgrounds (e.g., psychology, psychiatry, philosophy, sociology, and medical ethics) received the adapted version of the SACS and were asked to report on aspects of feasibility, such as duration of completion and comprehensibility of the items, as well as on the face validity of the items. Analyses on internal consistency and construct validity were conducted on the broad data collection.

### Data Collection

The data for the validation of the SACS were collected in three ways. Firstly, we conducted an online survey, which included our German version of the SACS and additional sociodemographic questions, among mental health professionals working in two psychiatric hospitals of the Regional Association of Westphalia-Lippe (LWL), a large mental healthcare provider in North Rhine-Westphalia, Germany. Mental health professionals, mainly nurses and doctors, received the link to the online survey *via* email and were invited to participate anonymously. Since the number of responses (*n* = 81) from this survey was insufficient, we looked for further recruitment strategies and asked all participants of a one-day conference (“*LWL-Fortbildungstag*”) to fill out a paper version of the questionnaire anonymously. On this occasion, we received *n* = 25 questionnaires from mental health professionals. As a third recruitment strategy, we visited multidisciplinary team meetings in four additional psychiatric hospitals in North Rhine Westphalia, of which two belonged to the Regional Association of Westphalia-Lippe and two to other healthcare institutions. Paper versions of the questionnaire from *n* = 103 mental health professionals were gathered here.

### Data Analysis

The analysis was conducted using IBM Statistics SPSS 26 and the results presented in this paper refer to the combined sample of *N* = 209. Sociodemographic variables were analyzed regarding descriptive aspects, such as measures of central tendency and variability. During the translation and adaptation doubts about the original factor structure arose, which will be further described in the results. These doubts indicated that it would not be sufficient to assess the goodness of fit of the existing empirical model (i.e., the original factor structure) with our data, but also an alternative model had to be provided, which would better represent the underlying structure of the items. For this reason, we conducted an exploratory factor analysis instead of a confirmatory factor analysis to be able to examine the item structure of the developed German version of the SACS in an unbiased way. Furthermore, the three-factor solution was specifically tested within the factor analysis to verify the original structure with three independent subscales. The total sample seemed to be sufficient for this analysis considering the common advice for sample sizes for factor analyses (56). Furthermore, the suitability of the data set for the following factor analysis was checked using the Kaiser-Meyer-Olkin criterion. The cut-off for the factor loadings was set at 0.4 (57), and Cronbach's Alpha was interpreted as acceptable when > 0.7, good when > 0.8 and excellent when > 0.9 (58).

## Results

### Translation and Adaptation

All English and German items can be found in Table 1, including their assignment to the subscales according to the original version of the SACS. Difficulties with the wording and content of some of the original items were observed during the process of translation and adaptation. Items that refer to two different aspects within one sentence are especially problematic. An example is item number 5, which associates coercion with both care and protection simultaneously, although care and protection are qualitatively different aims of coercive intervention. This difficulty also appears in the designation of the second subscale, which is called “coercion as care and security” and, thus, also addresses two different aspects simultaneously. Furthermore, some items refer to the same aspect but are merely conversely phrased, such as the items 6 (“more coercion should be used in treatment”) and 13 (“too much coercion is used in treatment”). Such conversely phrased items can be used to prevent biases when filling out the questionnaire. However, item 6 is assigned to the third subscale “coercion as treatment” and item 13 to the first subscale “coercion as offending.” The reason for this is unclear.

**Table 1:** Items of the original English version and the adapted German version of the SACS.

**Original subscale**	**Item**	**English wording**	**German translation**
Coercion as offending	3	Use of coercion can harm the therapeutic relationship.	Die Anwendung von Zwang kann der therapeutischen Beziehung schaden.
	4	Use of coercion is a declaration of failure on the part of the mental health services.	Die Anwendung von Zwang ist ein Zeichen für das Versagen des psychiatrischen Hilfesystems.
	8	Coercion violates the patients integrity.	Zwang verletzt die Integrität des Patienten.
	13	Too much coercion is used in treatment.	In der Behandlung wird zu viel Zwang angewandt.
	14	Scarce resources lead to more use of coercion.	Knappe Ressourcen führen zu mehr Anwendung von Zwang.
	15	Coercion could have been much reduced, giving more time and personal contact.	Zwang könnte stark reduziert werden durch mehr Zeit und persönlichen Kontakt.
Coercion as care and security	1	Use of coercion is necessary as protection in dangerous situations.	Die Anwendung von Zwang ist notwendig zum Schutz in gefährlichen Situationen.
	2	For security reasons, coercion must sometimes be used.	Aus Sicherheitsgründen muss manchmal Zwang angewandt werden.
	5	Coercion may represent care and protection.	Zwang kann Fürsorge und Schutz darstellen.
	7	Coercion may prevent the development of a dangerous situation.	Zwang kann die Entstehung einer gefährlichen Situation verhindern.
	9	For severely ill patients, coercion may represent safety.	Für schwerkranke Patienten kann Zwang Sicherheit darstellen.
	11	Use of coercion is necessary toward dangerous and aggressive patients.	Die Anwendung von Zwang ist notwendig bei gefährlichen und aggressiven Patienten.
Coercion as treatment	6	More coercion should be used in treatment.	In der Behandlung sollte mehr Zwang angewandt werden.
	10	Patients without insight require use of coercion.	Patienten ohne Einsicht benötigen die Anwendung von Zwang.
	12	Regressive patients require use of coercion.	Regressive Patienten benötigen die Anwendung von Zwang.

### Feasibility and Face Validity

Participants of the pre-test reported that all items were understandable and that it was feasible to fill out the adapted version of the SACS efficiently on their own. Regarding face validity, most professionals and researchers in the pre-test claimed that items seemed to address cognitive attitudes about the use of coercion.

### Sample Characteristics

Regarding the sociodemographic aspects, our total sample consisted mainly of nurses, while doctors and other members of the multi-professional healthcare team, such as psychologists, were less represented. Mental health professionals had an average of *M* = 14.88 (SD = 11.86) years of work experience within psychiatric institutions. In accordance with that, over 90% had already experienced situations in which coercive measures had to be applied. While over three-quarters of the sample had also participated in de-escalation training, only half of the participants had additionally attended training or conferences about the use of coercion in psychiatry. Further sociodemographic information of the sample can be found in Table 2. Ratings of the participants as means and standard deviations for each item can be found in Table 3.

**Table 2:** Sociodemographic characteristics of the total sample.

	***n***	**%**
**Group of age (*****n*** **=** **208)**		
Up to and including 25 years	17	8.2
26–35 years	70	33.7
36–45 years	31	14.8
46–55 years	60	28.6
56 years and over	30	14.3
**Gender (*****n*** **=** **208)**		
Female	87	41.8
Male	121	58.2
**Professional group (*****n*** **=** **207)**		
Nurses	151	72.9
Doctors	24	11.6
Psychologists, social workers and co-therapists	25	12.1
Other members of mental healthcare teams	7	3.4
**Previous experiences with the application of coercive**		
**interventions (*****n*** **=** **202)**		
Yes	189	93.6
**Participation in the additional training on the application of coercive**		
**interventions (*****n*** **=** **209)**		
Yes	111	53.1
**Participation in de-escalation training (*****n*** **=** **208)**		
Yes	169	81.3

**Table 3:** Ratings of participants (mean, standard deviation, range) for each item.

**Item number**	**Range (min-max)**	***M***	**SD**
1	1–5	4.01	0.96
2	1–5	4.11	0.82
3	1–5	3.91	1.06
4	1–5	2.28	1.07
5	1–5	3.72	0.90
6	1–5	1.81	0.92
7	1–5	3.52	1.12
8	1–5	3.68	0.98
9	1–5	3.64	1.01
10	1–5	2.42	1.02
11	1–5	3.70	1.09
12	1–5	2.35	0.85
13	1–5	2.80	1.00
14	1–5	3.58	1.20
15	1–5	4.04	0.96

### Reliability (Internal Consistency) and Construct Validity

**Figure 1:**
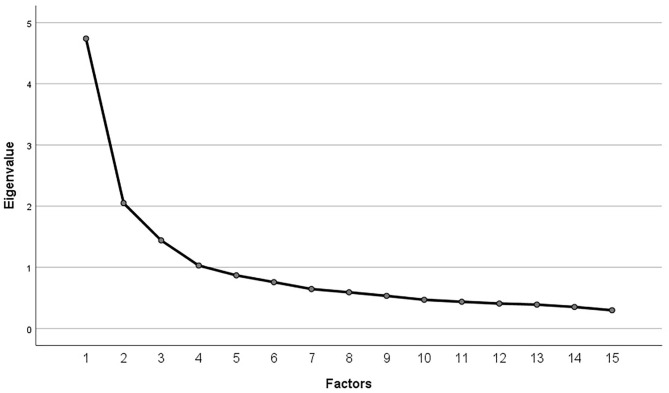
Scree plot of explorative factor analysis for all items of the German SACS.

The Kaiser-Meyer-Olkin value for the 15 items of the German SACS was 0.828, indicating that the sample was appropriate for conducting the factor analysis. The results from the anti-image correlation further showed only values higher than 0.75 on the diagonal. Thus, all items were suitable. The initial solution revealed four factors with Eigenvalues higher than 1, with the first factor having an Eigenvalue higher than four. The Eigenvalues for all factors can also be found in the scree plot in Figure 1. Further factor solutions were examined as the curve of the scree plot and loading of the items within the rotated component matrix did not support the initial solution.

Firstly, the original structure consisting of three factors and, secondly, a single-factor solution, as indicated by the Eigenvalues and the scree plot, were analyzed. Factor loadings for all items for both solutions can be found in Table 4. As can be seen, the three-factor solution represented mainly the original structure but with some items not loading clearly on one factor or, conversely, two factors. Furthermore, not all items loaded on the same factor as in the original structure. Internal consistency in the form of Cronbach's alpha of these subscales was merely sufficient, with α = 0.76 for the first factor, α = 0.762 for the second factor, and α = 0.76 for the third factor.

**Table 4:** Factor loadings in (rotated) component matrix for three-factor and single-factor solution.

	**Three-factor solution**			**Single-factor solution**
**Number of item**	**Factor 1**	**Factor 2**	**Factor 3**	**Factor 1**
1	**0.71**	−0.11	0.27	**0.65**
2	**0.75**	0.09	0.12	**0.48**
3	−0.08	**0.54**	−**0.44**	−**0.59**
4	−**0.51**	**0.56**	0.09	−**0.55**
5	**0.71**	−0.17	0.09	**0.58**
6	0.19	−0.27	**0.65**	**0.64**
7	**0.62**	−0.06	0.17	**0.51**
8	0.03	**0.63**	−0.36	−**0.52**
9	**0.63**	−0.09	0.26	**0.59**
10	0.19	−0.13	**0.78**	**0.65**
11	0.36	0.04	**0.66**	**0.59**
12	0.14	−0.13	**0.76**	**0.60**
13	−0.21	**0.71**	−0.07	−**0.55**
14	0.01	**0.76**	−0.04	−**0.42**
15	−0.03	**0.71**	−0.11	−**0.46**

*Bold values indicate those ≥ the cut-off set for factor loadings*.

The second solution with only one factor was also examined and, as can be seen in Table 4, the factor loadings speak strongly for one general factor with two opposite poles, as all items load highly either positively or negatively on this factor. All items of the original subscale “coercion as offending” loaded negatively on this factor, while all other items (of the original subscales “coercion as care and security” and “coercion as treatment”) loaded positively on this factor. All items loading negatively on the factor were conversely recoded to calculate the internal consistency of this scale. The scale revealed a high internal consistency with Cronbach's alpha α = 0.84.

## Discussion

### Empirical Validation

Difficulties already appeared with the wording of the items of the SACS and the aspects they referred to during the translation and adaptation of the original items. It could be recognized, *inter alia*, that some items refer to the same aspect but in a conversely phrased way. Some items apparently seem to belong to more than one of the original subscales and can, thus, be neither translated nor interpreted by mental health professionals in a clear way. Such difficulties represent general problems in the process of developing self-reported questionnaires. This can result in ambiguous answers and, consequently, also affect the item structure (59).

The validation of the original version (47) had already revealed items that loaded on two factors simultaneously, either in the same direction or in a converse way. Moreover, the basic assumption of three independent kinds of attitudes was revealed to be problematic. Independent factors, as proposed in the original validation study, would imply that staff members could have a critical, pragmatic and positive attitude simultaneously. Such a finding would only make sense if the attitudes assessed were not mutually exclusive, as is the case in the assessment of personality traits, such as the Big Five, as measured by the Neuroticism-Extraversion-Openness Five-Factor Inventory (60). In contrast to the Big Five (extraversion, agreeableness, openness, conscientiousness, and neuroticism), the attitudes assessed by the SACS are mutually exclusive inasmuch as it does not make sense that mental health professionals have a positive and a critical attitude toward coercion at the same time. Consequently, the original distinction between critical, pragmatic and positive attitudes toward the use of coercion cannot be maintained as independent simultaneous attitudes.

Instead, the wording of the items and our results seem to predominantly justify one factor representing two opposite poles, ranging from a rejecting to an approving attitude toward the use of coercion. Based on our results, staff members could be categorized into three groups (rejecting coercion, approving coercion, or ambivalent) according to their value on this scale. Interestingly, the results of this classification correspond to the initial results from the research of Husum et al. (47), which could identify these three groups in a focus group with mental health professionals. It also reflects the study of Alem et al. (61), which was the source of inspiration for Husum et al. for the item construction of the SACS. The study by Alem et al. operationalized attitudes toward coercion as the tendency of mental health professionals to view coercive interventions as ethical or unethical. This would strengthen the idea that mental health professionals can be classified according to their cognitive attitudes about the use of coercion. The important differences lie in the concrete assessment and generation of these categories or groups.

### Further Implications

From a conceptual perspective, it can be observed that all items on the SACS measure the staff's beliefs about coercion, and particularly that no item measures their emotional dispositions toward coercion. Consequently, the SACS focuses exclusively on the cognitive components of staff attitudes to the neglect of emotional components. This is problematic, inasmuch as it can reasonably be expected that emotional components of staff attitudes will play a role in the use of coercion (54) as coercive situations are also associated with strong emotions in the staff involved. Furthermore, reasons to use coercive measures might also be of an emotional quality, for example, anger or fear as a result of aggressive behavior or compassion toward the patient.

The development of the original SACS (47) was an important step toward a validated assessment of mental health professionals' attitudes toward coercion and prompted important research on coercion in psychiatry in various countries in the past few years. From a methodological perspective, our findings have implications for results from previous research relying on the original three subscales of the SACS (31, 48–50, 52–54). The results of these studies should be interpreted with caution, as they might not be able to be maintained. From an ethical perspective, the use of unvalidated scales to measure staff attitudes toward coercion is problematic, insofar as it may yield unjustified hypotheses about which strategies might be effective in reducing coercion and, thus, pose an obstacle to evidence-based practice.

Further research on staff attitudes toward coercion is important because it can yield useful hypotheses for the development of strategies to reduce coercion and studies that test their effectiveness. This research, in turn, can inform the priority setting in the reduction of coercion. Researchers using a validated version of the SACS should be aware of the restriction that the SACS assesses explicit cognitive attitudes exclusively. Recent research, though without using validated measurements, suggests that emotions might also be relevant in this context (54). Validated instruments to measure attitudes toward coercion that encompass cognitive, emotional and behavioral aspects should be developed and used to examine their role in the use of coercion in psychiatry. This would make the development of more focused strategies to reduce coercion possible. If future research, for example, were to find that emotional attitudes play a bigger role in the use of coercion than cognitive attitudes, it would make sense to shift the focus of professional training away from forging cognitive change (e.g., by providing information about the criteria of using coercive measures) toward forging emotional change (e.g., by inviting a peer support worker or a service user to share his or her personal experiences of coercive measures).

### Strengths and Limitations

The key strength of our study is the fact that it is, to the best of our knowledge, the first empirical validation of a German version of the SACS. Moreover, our approach to the review of the original version and the interpretation of the empirical data is broader than usual in validation studies as we also discussed conceptual and ethical implications. Regarding limitations, it must be stated that we only recruited mental health professionals in one German state: North Rhine Westphalia, which limits our data to a rather specific sociocultural and legal context. Additionally, our sample was skewed, as most of our participants were nurses and we only had a small proportion of doctors and other members of the multi-professional mental healthcare team. However, the sample size was comparable to the validation study of the original version (47) and seems to be representative of the clinical reality, as nurses represent the largest professional group in psychiatric hospitals in Germany. Furthermore, nurses are usually directly involved in situations in which coercive measures are applied and are, therefore, highly relevant for the topic examined in our study.

## Conclusion

We provide a validated German version of the SACS in our study. All items of the original version could be adequately translated into German and it could be verified that they are comprehensible and suitable for mental health professionals working in German psychiatric institutions. Even though our validation did not replicate the original factor structure (47) consisting of three independent subscales, it did reveal a single-factor solution with good internal consistency. Therefore, the German version of the SACS enables researchers to assess staff members' explicit cognitive attitudes toward the use of coercive measures in mental health services in German-speaking countries in a self-reported and validated way.

Wider methodological and ethical conclusions can be drawn from the results presented. Our study highlights the importance to reflect critically on the use of unvalidated instruments in research, especially when these results are used as the basis for the development of clinical interventions (e.g., to reduce the use of coercion). If results from empirical studies are used as premises in ethical debates, foundations of clinical interventions or models of care, those results should be free of biases and methodological difficulties.

## Data Availability Statement

The datasets generated for this article are not readily available because they contain data that has not been analyzed and published yet. Upon reasonable request, they will be made available by the corresponding author. Requests to access the datasets should be directed to Simone A. Efkemann, simone.efkemann@rub.de.

## Ethics Statement

The studies involving human participants were reviewed and approved by the Research Ethics Committee of the Medical Faculty of the Ruhr University Bochum. The participants provided their informed consent to participate in this study.

## Author Contributions

SE, RB, GJ, and JG designed the study. SE wrote the protocol. SE and JG managed the collection and preparation of data. SE, MS, and JG conducted the analysis and interpretation of data. SE wrote the first draft of the manuscript. MS and JG corrected and edited the manuscript. All authors contributed to and have approved the final version of the manuscript.

## Conflict of Interest

The authors declare that the research was conducted in the absence of any commercial or financial relationships that could be construed as a potential conflict of interest.
